# Which Would Be Right?

**Published:** 1874-07

**Authors:** H. Scott


### WHICH WOULD BE RIGHT?

BY H. SCOTT.

There are yet, nevertheless, occasionally cases presented to the most experienced of us that cause us to hesitate as to the the right thing to be done. A month ago a mother brought her little girl of five years to have a tooth extracted. It was the second right inferior molar. It was an uncommoly large tooth. It had been aching several days and nights, and paliatives had ceased to mitigate the pain. There was no indication of incipient periostitis. I suggested further treatment, but the mother insisted on having the tooth out, and I complied. The fangs were frightfully divergent, long and turned, requiring great force to bring them away, and lacerating the gums very much in doing so all of which frightened the child painfully.

Two days since the child was brought back with the corresponding tooth of the left side in a similar condition. I refused to extract it, and insisted on treatment; but the Irish mother was inexorable, and said she would take the child to another dentist if I would not pull it. It had been aching and keeping them all awake night after night, and her husband said it must come out; I took it out. It was more formidable than the first, and brought with it an inch of the external alveoli. There was frightful hæmorrhage and laceration of the gums. Nothing unusual followed, and all is right now.

What shall we do in such cases? Would it be proper to devitalize the pulp with arsenic, with the risks of subsequent consequent inflamation? or where our suggestions are not accepted, should we allow the case to fall into other hands where we had not much confidence. Is it right to use arsenic in the deciduous teeth at all? and is it right to extract them from three to five years previous to the time of eruption of the permanent ones? In this case both molars were decayed down to the pulp, leaving little above the gums besides enamel; and the family is one that would give no attention to teeth beyond extracting them.

